# Editorial

**Published:** 1915-10

**Authors:** 


### EDITORIAL

The Atlanta Journal-Record of Medicine has its Business Office at 23 West Alabama Street where all business communications should be sent.

Remittances are payable to The Journal-Record of Medicine.

Concerning Original Communications and Editorial matters, address Richard R. Daly, M. D., 708 Empire Life Building, Atlanta, Ga.

Reprints of Original Articles will be furnished at cost price. Requests for them had best be made on the Manuscript.

Upon request, we will present, postpaid, to each contributor of an original article, twenty copies of The Journal-Record of Medicine containing such article.

### DR. REGISTER HEADS AMERICAN MEDICAL EDITOR'S ASSOCIATION.

At the meeting of the American Medical Editors' Association held in New York City October 18 and 19, Dr. Edward C. Regi ister, Editor of The Charlotte Medical Journal, was elected President of the Association.

No better selection could have been made, for Dr. Register has had long experience in the management of medical publications and his skill in discerning the valuable things in scientific literature and his facility in presenting them has been demonstrated in his work, and his enthusiasm for co-operation will be of utmost assistance in successful administration of the Association's affairs. His personal qualifications are such as to endear him to his fellows and to bring to the next meeting the best they have to offer.

Dr. H. Edwin Lewis, the retiring President and Editor of American Medicine, said in his address that the medical publications had great power for good among the people through the influence they wield with the profession and that a careful observation and exercise of this great force can be of untold service. That this power has been placed under the inspiration of Dr. Register speaks well for the judgment of the Association and for the future of all good and substantial medical journals. Both the Association and Dr. Register are to be congratulated upon the event.

There were many excellent papers and speeches at the meeting and the suggestions made and elaborated were invaluable.

---

### PREPAREDNESS IN SURGERY AND MEDICINE.

We have advanced a great way in the care of many diseases since the Spanish war and we like to think that there could be no recurrence of the domestic camp troubles and mortality that marked that deplorable period. But are we at all sure of ourselves in regard to active warfare either in this country or upon foreign soil? The military people have shown that we are far from ready in that regard and the attitude of large numbers of people is against preparation because it may lead to war itself. That we are not really ready as medical men goes without saying when we read at all carefully of the conditions to be met with in the armies of Europe and the immense and horrid problems the surgeons have to solve. The mortality is wonderfully low, it is true, and even typhus has been controlled, but there are other conditions that have scarcely been touched and which hamper the surgeons greatly.

Just now the matter of supply itself would be hard to control when drugs are growing scarcer and some of them disappearing from the market. We are dependent upon the fighting nations for materials they need themselves and can not send to us because of blockades besides. Warlike affiliations might arise so as to prevent our getting our drugs even if we were not fighting the drug growers directly. The nation should encourage in every way its self-reliance in the necessities. No matter what the increased price be for a time, we should insist upon Made-in-America being the mark of all our supplies and then attend to it that though we gave a monopoly to home products, we demanded the best the world could supply.

The cost would not be any greater than we are paying now with quinine at about $2.50 instead of .58 per ounce, and bromide of soda at $4.00 instead $1.90 a pound, while numerous other drugs have similar rises in price and we have to pay for them whether we are at war or not. All these can be handled primarily in our own country, barring of course such things as grow in the tropics. It is the manufacture that counts.

But not alone with materials are we to be concerned and deeply so. The men themselves who are competent to do the great work and to met the peculiar demands of warfare are not to be found everywhere. Special training is as necessary in that regard as in any other where skill counts for success and we commend it to the great societies of the country to make some organization leading to a preparation for war that will be commensurate with that of the army and navy so far as the work of the people is concerned.

---

### ATTENDING MEETINGS.

Not only have the great medical societies of the country a certain responsibility in the matter of leading the profession to see and do the large things the nation demands, not only do they afford opportunity for men to get a better social grasp upon each other's personalities, but they afford a stimulus that is psychic in character and one that is so easily recognizable as to be cultivated with profit.

The men who are doing big things are probably more conscious than anyone else of the time when they were not doing them. They know of the needs that attracted their attention and of the weary time spent in hunting for a solution of these needs before their study was rewarded. They know also that even with the best they have to offer and do there are still demands that are not fully met. The big men are the broad-minded and meetable chaps that anyone and everyone competent to receive their messages can question personally and be sure of a courteous and careful reply. They know too much to bother about themselves and they recognize that the big things in the world are common property no matter who first discovered them. They are ready to show the riches in their custody and to let everyone share according to his own capacity.

The Medical societies large and small give the opportunity to meet such men when they are members. Attendance upon meetings where men tell simply of the wonderful things done and try to show to all that the value is to be universal is a peculiar inspiration to a listener and when he knows that he has only to step forward to have every little thing told to him, the experience is so invigorating that the societies are accounted for.

Even in the smaller societies of the counties, it is remarkable how often there are collated by the speakers in possibly accidental apposition the canditions that expose and discover new aspects of disease.

The Medical Societies have done marvellous things for the profession and they have wonderful opportunities opening before them to do as great things for the people at large.

---

### NEW MEDICAL JOURNAL.

The Journal of Laboratory and Clinical Medicine, published by The C. V. Moseby Company of St. Louis, having an able staff of editors headed by Dr. Victor C. Vaughan, of the University of Michigan, makes its bow to the medical public in its initial issue for October.

Its announced purpose is to bring the laboratory men and the clinicians closer together by giving information valuable to each and so arranging it as to be readily comprehensible.

The first number has an excellent program and is interesting throughout.

The press and book work are of fine quality as is to be expected from the publishers.

We welcome the new Journal's advent and feel sure that its place in the sun will be securely occupied.

